# The Involvement of Past Traumatic Life Events in the Development of Postpartum PTSD after Cesarean Delivery

**DOI:** 10.3390/healthcare10091761

**Published:** 2022-09-13

**Authors:** Eirini Orovou, Maria Dagla, Panagiotis Eskitzis, Georgios S. Savvidis, Nikolaos Rigas, Alexandros Papatrechas, Angeliki Sarella, Christiana Arampatzi, Evangelia Antoniou

**Affiliations:** 1 Department of Midwifery, University of Western Macedonia, 50100 Kozani, Greece; 2 Department of Midwifery, University of West Attica, Agioy Spyridonos 28, 12243 Egaleo, Greece; 3 Department of Occupational Therapy, University of Western Macedonia, 50100 Kozani, Greece; 4 Non-Profit/Non Governmental Organization (NGO) “Fainareti”, 12243 Athens, Greece

**Keywords:** postpartum PTSD, PTSD profile, cesarean section, cesarean delivery, traumatic life events, birth trauma

## Abstract

Background: Although childbirth is considered a natural process, a high percentage of postpartum women consider it traumatic. Any previous traumatic event in a woman’s life can be revived through a traumatic birth experience, especially after a complicated vaginal delivery or cesarean delivery. The purpose of this study was to clarify the relationship between previous traumatic life events and posttraumatic stress disorder (PTSD) in postpartum women after cesarean section and which specific events exerted the greatest influence. Methods: A sample of 469 women who had undergone cesarean sections at a Greek university hospital consented to participate in this prospective study. Data from a medical/demographic questionnaire, life events checklist, perinatal stressor criterion A, and posttraumatic stress checklist were used to evaluate past traumatic life events and diagnose postpartum posttraumatic stress. Results: Out of 469 women, 25.97% had PTSD and 11.5% a PTSD profile, while 2.7% had PTSD and 2.7% a PTSD profile. Also, it appeared that only specific direct exposure to a traumatic event and/or witnessing one were predictors of postpartum PTSD. Conclusions: This survey identified specific traumatic life events, psychiatric history, stressor perinatal criterion A, preterm birth, and emergency cesarean section as risk factors for the development of PTSD or a PTSD profile in women after cesarean delivery.

## 1. Introduction

In a person’s life, many unpleasant events can occur, more or less significant, with a different impact each time on themselves and their environment. However, when can an unpleasant event be characterized as traumatic? The characteristics that place an event in the category of trauma consist of: (a) the unexpectedness of an event that upsets the individual’s mental world, (b) an unusual event that takes the individual by surprise, (c) reduced ability to control the event that generates a feeling of weakness, and (d) irreversible long-term consequences, which exhaust the mental resources available to the person [1,2]. Behavioral reactions vary, and may include exhaustion, anxiety, sadness, shallow emotion, agitation, and even withdrawal from daily activities, and may last from weeks to months until the person returns to their previous state. In more severe cases, there is continued distress without periods of respite, intense dissociative symptoms, and intense reliving of the event despite the person’s return to safety. However, the somatization of symptoms indicates an inability to express emotional distress and is usually a sign of serious mental illness [3]. The majority of people who have experienced trauma report feeling better gradually after the event. This is because most people develop appropriate coping strategies, including social support, to deal with the aftermath of the traumatic event [3]. However, if after one month the symptoms get worse instead of going away, the person may be suffering from posttraumatic stress disorder (PTSD) [4,5].

According to the fifth edition of *Diagnostic and Statistical Manual of Mental Disorders* (DSM-5) [6], PTSD can occur after someone goes through a life-threatening event, such as disaster, assault, combat, rape or other sexual violence, threats of death, or terrorist attack [3,7,8]. The National Center for PTSD calculates that approximately of 8% of the general population will have PTSD at some point in their lives [9]. However, women are twice as likely to develop PTSD than men [10], and this reflects several stressful situations, hormonal changes, domestic violence, and traumatic childbirth experiences that affect females [11]. In addition, after a traumatic event, women blame themselves more often than men, as a result of which they have an increased chance of experiencing depressive feelings, becoming victims of addictions (drugs, alcohol) or developing PTSD [12].

### 1.1. Past Traumatic Life Events and PTSD

Any previous PTSD can influence its reappearance. Several studies report at least one to four traumatic experiences in individuals with PTSD [13,14]. The more and more severe the past traumatic events are, the greater the expected development of PTSD [15]. Therefore, individuals who have had a traumatic experience in the past and developed PTSD are at greater risk of redevelopment of the disorder through sensitization [16].

### 1.2. Birth Trauma and PTSD

Although childbirth is considered a natural process, 19–45% of postpartum women consider it traumatic [17]. Under certain circumstances, atraumatic birth experience can lead to postpartum PTSD [18]. The incidence of postpartum PTSD after a traumatic birth is about 19% and much higher than that in the general postpartum population [19].

PTSD after childbirth was first investigated by Menage in 1993 [20] and involved women undergoing obstetric procedures. The findings showed a new cause of PTSD that had not been reported before, which was beginning to challenge the practices applied until then in obstetrics, including the emotional weakness of women during childbirth, the personal experience of pain, the lack of information provided, and the behavior of health professionals. The findings of another study published in 1995 [21] showed a difficulty in developing the mother–child bond and additional depressed mood in mothers with a traumatic birth experience. Since then, research on women with traumatic birth experiences has increased, and it is now known that certain birth experiences can lead to postpartum PTSD [22,23]. Several factors seem to be responsible for the development of the disorder, such as pathology of gestation, fear of childbirth, complications during delivery, specific mental disorders, low partner support, inclusion of a neonate in a neonatal intensive care unit (NICU), and past traumatic life events in the woman’s life [23,24,25,26,27]. When the mother has experienced previous traumatic events, it is quite difficult to determine the cause of postpartum PTSD. Since previous traumatic events can be recalled and trauma symptoms appear, a reexperiencing of an old untreated PTSD can coexist with the new birth trauma [23,24,26,28].

### 1.3. Type of Cesarean Section and PTSD

The relationship between the type of delivery and the development of PTSD has been studied by many researchers [29,30,31,32]. However, there are several previous and new studies that identify the emergency cesarean section (EMCS) as a major factor in the development of PTSD [22,26,28,30,33], although some others [34,35,36] do not differentiate between the effect of EMCS and elective cesarean section (ELCS) in the development of posttraumatic symptomatology and conclude that the development of PTSD after CS is due to other factors [37]. More specifically, several studies report that some psychosocial factors, such as previous history of mental disorders, lack of support from the partner, and previous traumatic events, have a strong correlation with postpartum PTSD [24,26,38,39].

The purpose of this study was to clarify the relationship between previous traumatic life events and PTSD in postpartum women after CS and which specific events exerted the greatest influence.

## 2. Materials and Methods

This study took place from July 2019 to June 2020 at the Obstetrics Department of the General University Hospital of Larissa in Greece. The Ethics Commission of the University Hospital of Larisa approved this research: 18838/08-05-2019. To achieve the purpose of this study, a prospective design was used.

### 2.1. Study Participants

Our first sample consisted of 490 women who had undergone CS at the specific hospital, and gave written consent for participation in the study. In sum, 469 women replied to the follow-up and constituted our sample.

### 2.2. Inclusion and Exclusion Criteria

Inclusion criteria for women to participate in the research were: (a) having given birth with CS, (b) having been monitored throughout or during most of their pregnancy in the hospital such that their medical file was complete with information, (c) adequate understanding of the Greek language, (d) adequate mental level, and (e) not having taken psychotropic drugs or substances during the specific period. Those who did not meet the above criteria were excluded from the study.

### 2.3. Data and Measures

The data were collected at two time points. On the second day after the CS, in the Midwifery Department, self-administered questionnaires (sociodemographic, Life Events Checklist criterion A), were given by the researcher to postpartum women after their informed consent, while information regarding medical history was recorded from each woman’s medical file. During the sixth week postpartum, via telephone, women answered questions on the postpartum PTSD scale. This specific time was selected in order to meet the appropriate period of over a month to establish the diagnosis of PTSD [3]. All measures were created by the National Center of PTSD Staff [9] as per the DSM V [6], and were translated and weighted into the Greek language by the research team.

#### 2.3.1. Sociodemographic Questionnaire

This questionnaire included sections on social, demographic, medical (history, obstetric, gynecological, psychiatric, and neonatal) and questions about birth trauma after CS.

#### 2.3.2. Life Events Checklist for DSM-5 (LEC-5)

This measure is designed to screen past traumatic life events [40]. The LEC-5 estimates exposure to 16 traumatic events that probably led to PTSD or distress, and also contains another item that assesses a stressful event not captured by the previous questions. The LEC-5 does not have a scoring protocol, so it is limited to identifying the traumatic events. A very important feature of the LEC-5 is that respondents indicate different levels of exposure to each type of traumatic event; therefore, each individual can have multiple levels of exposure to an event (happened to me, witnessed it, learned about it, part of my job, not sure, does not apply) [41].

#### 2.3.3. Stressor Perinatal Criterion A Based on DSM-5

Criterion A expresses the type of traumatic event from which the remaining symptom clusters of reliving, avoidance, negative mood changes, and hyperarousal may appear [6]. A necessary condition to meet criterion A is the individual’s exposure to an actual or threatened death, serious injury, or sexual violence through one of the different levels of exposure: (1) direct exposure, (2) witnessing the event, (3) exposure through information that the event happened to a loved one, and (4) exposure to unpleasant details of an event [42].

However, for the needs of this study, criterion A had to examine exposure in the special population of women after EMCS and ELCS. Therefore, it was adapted to the specialized requirements by formulating appropriate questions. (1) During the CS, did you feel that your life or the life of your child was in danger? This question has four possible answers (one affirmative for the life of the mother, one affirmative for the life of the child, one negative and one negative concerning the lives of both). (2) Have there been any serious complications with your health or your child’s health? This question has three possible answers (one affirmative for the mother, one affirmative for the child and one negative). Stressor perinatal criterion A was divided into criterion A1, which concerns life-threatening situations of the mother or child during or shortly before CS, and criterion A2, which concerns complications in mother–child life.

#### 2.3.4. Posttraumatic Stress Checklist for DSM-5 (PCL-5)

This is a 20-item self-report scale that assesses 20 symptoms of PTSD according to the DSM-5 [43]. The PCL-5 offers a provisional diagnosis of PTSD. Individuals are asked to respond on a 5-point scale—0 (not at all), 1 (a little), 2 (moderately), 3 (quite a bit), 4 (very much)—on 20 self-report items assessing 20 symptoms of PTSD of the criteria: B (reexperiencing), C (avoidance), D (negative thoughts and feelings), and E (arousal and reactivity).

If criteria B, C, D, and E are met in connection with stressor criterion A, the provisional diagnosis of postpartum PTSD is considered certain [16]. In order to define the severity of the posttraumatic symptoms, the sum of the score of all answers with a PCL score ≥33 is also calculated [44]. If the score is <33 or if not all criteria from the PCL-5 are met, the incident is classified as a PTSD profile. This subtype of PTSD includes the most important PTSD symptoms without meeting all the diagnostic criteria to make a complete diagnosis [45]. However, the PTSD profile is associated with high levels of social and occupational morbidity (frequent absence from the workplace), suicidal ideation, alcohol use, and increased use of health-care services, resulting in significant impairment in the individual’s social life [37,46].

### 2.4. Statistical Analysis

Quantitative variables are expressed as mean values (SD) or as median values. For comparisons of proportions, chi-squared and Fisher’s exact tests were used. Logistic regression analyses were performed to identify traumatic events associated with the presence of PTSD or PTSD profile. Unadjusted and adjusted odds ratios with 95% confidence intervals were computed from the results of the logistic regression analyses. Statistical significance was set at 0.05 and analyses were conducted using SPSS statistical software (SPSS Statistics version 22.0, IBM, Armonk, NY, USA).

## 3. Results

Data from 469 women who had had a CS with a mean age of 32.58 ± 6.15 (*SD*) years, were analyzed. The ELCS proportion was 61.4% (*N* = 288), while 38.6% (*N* = 181) had an EMCS. Age, nationality, family, and financial status, as well as medical history, were similar in women with EMCS and ELCS postpartum women.

Table 1 presents the results of a chi-squared test showing relationships between the type of CS (independent variable) and diagnosis (dependent variable).

**Figure 1:**
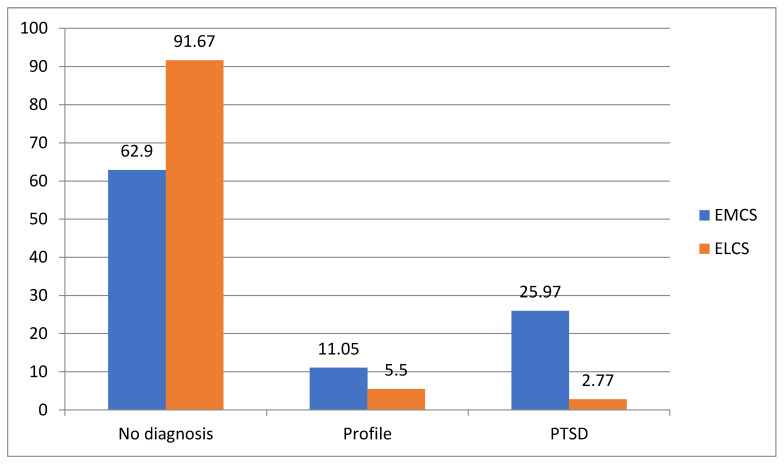
Graphic representation of the proportion of the participants with PTSD profile and PTSD according to type of CS.

The proportion of the participants who underwent an EMCS with a profile of PTSD was 11.05% and 5.56% in women with ELCS, while the corresponding proportions for having PTSD were 25.97% for EMCS and 2.7% for ELCS women (Figure 1). Consequently, the participants who had EMCS were more likely to have a PTSD profile and/or PTSD than those with ELCS.

For almost all independent variables presented in Table 2, and PTSD as dependent, one-way analyses of variance (ANOVA) were applied.

Taking into consideration perinatal and mental health variables from Table 2, statistically significant relationships with PTSD can be noted. Multiple analyses revealed that gestational week, type of CS, birth expectations, psychiatric history, traumatic CS, and criterion A1 and A2 variables were independently associated with the presence of PTSD.

Table 3 presents the results of a linear regression analysis investigating the relationship between PTSD and the LEC-5 categories of response to a traumatic event, in terms of all four LEC-5 categories, as if they were all potential predictors for PTSD.

Through linear regression, with PTSD being the dependent variable, it appeared that only direct exposure to a traumatic event (β = 0.41, *p* < 0.001) and witnessing one (β = 0.17, *p* = 0.019) were predictors of PTSD. The regression equation reached significance (F = 34.35, df = 4, *p* < 0.001, R = 0.228 and R^2^ = 0.222), showing that 22.2% of PTSD variance could be explained by this regression model. Accordingly, the analysis revealed that direct exposure and witnessing a traumatic event were associated with PTSD, revealing in this way the multiple meanings of the PTSD concept.

Taking into consideration the results of the previous analysis, for this study, only the “direct exposure” and “witness to the event” responses were summed in order to create an index of history of traumatic life events. Each item of the LEC-5 list was dichotomized, assigning a score of 1 only if the respondent gave one of the two abovementioned responses, and a 0 was assigned for either of the two remaining responses. To investigate the relationship between the 17 LEC-5 traumatic life events as potential predictors for PTSD, another linear regression analysis was applied (Table 4).

The present linear regression model introduced six predictors that were strongly related to PTSD: physical assault (β = 0.13, *p* = 0.006), assault with a weapon (β = 0.20, *p* = 0.039), life-threatening illness/injury (β = 0.09, *p* = 0.027), severe human suffering (β = 0.18, *p* < 0.001), sudden violent death (β = 0.16, *p* = 0.007), and other very stressful experience (β = 0.11, *p* = 0.011). Again, the regression equation reached significance (F = 10.94, df = 17, *p* < 0.001, R = 0.292 and R^2^ = 0.265). The explained proportion of PTSD variance by this model was 26.5%. Therefore, participants that had experienced any of the traumatic events appearing as a predictor in the present regression model were more likely to develop PTSD.

The last linear regression analysis in this study was applied to explore the relationship between the 17 LEC-5 traumatic life events and PTSD profile as dependent variable. The results are presented in Table 5.

As with the previous one, this linear regression analysis revealed sixpredictors for PTSD profile, but different from those relating to PTSD: transportation accident (β = 0.12, *p* = 0.011), serious accident at work/home/during recreation (β = 0.11, *p* = 0.026), assault with a weapon (β = 0.23, *p* < 0.001), sexual assault (β = 0.11, *p* = 0.033), captivity (β = 0.16, *p* = 0.010), and life-threatening illness/injury (β = 0.17, *p* < 0.001), the latter being the only common predictor in both cases. The regression equation reached significance (F = 6.46, df = 17, *p* < 0.001, R = 0.217 and R^2^ = 0.184), indicating that 18.4% of PTSD variance profile could be explained by this regression model. Finally, participants who had experienced any of the abovementioned traumatic life events have a bigger likelihood ofdeveloping a PTSD profile.

## 4. Discussion

The purpose of this study was to clarify the relationship between previous traumatic life events and PTSD in postpartum women after CS and which specific events exerted the greatest influence. The results showed that from 181 (36.6%) women who underwent an EMCS, 25.97% had PTSD and 11.05% a PTSD profile. From 288 women (61.4%) who underwent an ELCS, 2.79% had PTSD and 5.56% a PTSD profile. Birth expectations were not met for 52.2%, while 51% characterized their CS as a traumatic experience. Moreover, it was found that postpartum PTSD after CS was associated with EMCS, preterm birth, unfulfilled birth expectations, presence of the stressor perinatal criterion A, psychiatric history, and specific traumatic life events.

There are several studies that identify the EMCS as a traumatic birth experience and a major cause of postpartum PTSD [28,47,48]. For instance, a study published in 2012 [49] indicated high levels of PTSD (43.8%) in women after EMCS compared to ELCS (23.2%) and other type of deliveries. On the contrary, some other articles [24,37] maintain that some psychosocial factors are linked with postpartum PTSD. The reason that makes women with EMCS more vulnerable to developing PTSD is the emergency nature of the surgery.

The experience of EMCS is, of course, a traumatic event that differs from common acute traumatic events in the following parameters. (a) Presence of the stressor perinatal criterion A: In this case, specific criterion A includes immediate exposure to a threatened death or threat to physical integrity (hemorrhages, preeclampsia, eclampsia), but also exposure of the mother as a witness to events involving the fetus or neonate (fetal hypoxia, prematurity) [50]. (b) Inevitable symptoms of discomfort [50]: CS is major surgery, and there are usually complications of anesthesia in the first few hours, more pain and discomfort, and the mobility of postpartum women is affected [51]. Regardless of the type of CS, increased rates of surgery complications and also increased rates of rehospitalization have been observed [52]. Therefore, after CS, in addition to the physical discomfort, there is also mental discomfort that comes either from the existence of the unexpected event or from the inevitable symptoms of the surgery and recovery from it [50]. (c) Symptoms of hyperarousal. All the above postpartum period symptoms are very similar to the hyperarousal symptoms of PTSD. The postpartum period is characterized by a state of instability in emotions and mood, such as anxiety, insomnia, decreased appetite, and irritability. In addition, declining estrogen and progesterone levels and changes in melatonin levels have an effect on maternal circadian rhythms and thus irritability [53].

Another very important factor is the presence of past traumatic life events. Our results show that previous traumas of direct exposure and witnessing exposure were major factors in postpartum PTSD and PTSD profile, as opposed to informational exposure or exposure in the work environment. Another category of traumatic events that had a positive correlation with postpartum PTSD was the trauma of severe human suffering. Therefore, women’s previous experiences that included the possibility of the threat of serious injury or death, serious physical injuries, and serious life-threatening issues in a population (e.g., starvation) [54] seemed to be reviving during the woman’s postpartum period.

There was a significant link according to our results between traumatic events that occurred after physical assault or assault with a weapon and postpartum PTSD or a PTSD profile. A mother’s history of abuse, specifically childhood abuse, appears to have a significant impact on her mental health in the postpartum period and equally affects the development of the mother–child bond, according to Seng and her colleagues [55]. Several studies [56,57,58,59] have reported that intimate partner violence is an important factor in the development of postpartum PTSD and other perinatal mental disorders. Similarly, sexual abuse as a traumatic experience has been reported by other authors [60,61] and related to PTSD symptoms. Results of a meta-analysis reported that 75% of sexual assault survivors met the criteria for PTSD after one month [62]. One explanation for this phenomenon in perinatal period is that pregnancy and delivery process may activate the woman’s experiences of abuse. It seems that invasive examinations, the pain of childbirth, the feeling of losing control, and the unequal relationship between health professionals and woman can act as a trigger for the activation of the mechanism of the development of PTSD symptoms. In general, during childbirth, both the woman’s body and soul participate, and when she feels violated, she reacts faster to the new trauma [63].

Also important was the finding of our study that showed a statistically significant factor of captivity (kidnapping, hostage, or prisoner of war) for the development of PTSD symptoms after CS. Although there is no other research that studied the effects of war on childbirth, there are several cases of reported PTSD in war victims (soldiers or civilians) [64,65]. In addition, traumatic events that occurred after transportation accidents, accidents at work, at home, or during recreation were some of the groups of traumatic events that were associated with postpartum PTSD symptomatology.

Finally, a mediating role of psychiatric disorders (anxiety disorders, depression, psychosis, and history of postpartum mental disorders) in the development of postpartum PTSD emerged from the findings of the present study. Indeed, the mother’s history of psychiatric illness is recognized as a risk factor for the development of postpartum mental disorders [66], and several studies agree with our findings. In a systematic review published in 2012 [31], the maternal history of psychiatric disorders was one of the most validated risk factors for postpartum PTSD. Therefore, depression during pregnancy [67] previous PTSD [68,69], anxiety and depressive disorders in general [70,71], and tokophobia (as a specific anxiety disorder) [38] are predictive markers of postpartum PTSD or PTSD profile. An explanation for the positive relationship between psychiatric history and PTSD is the determining role of the existence of previous trauma. In more details, mental illness may account for exposure to trauma, and conversely, exposure to trauma may account for the development of a mental illness [72].

### Strength and Limitations

The major strength of this article is that is the first to investigate the relationship between past traumatic life events and postpartum PTSD in women after cesarean delivery. However, an important limitation of our study mainly concerns that it focuses only on women after cesarean section. Therefore, we do not know how a woman responds to psychological trauma after a traumatic vaginal birth. In future research, it will be good to examine how a past traumatic experience is revived through all types of childbirth.

## 5. Conclusions

The present study identified specific traumatic life events, psychiatric history, the stressor perinatal criterion A, preterm birth and EMCS as risk factors for the development of PTSD or a PTSD profile in women after cesarean delivery. The results of the study showed that for some women, the process of giving birth led to the revival of a traumatic experience, activating the mechanism for the development of a new trauma. Therefore, it is deemed necessary to create the appropriate birth conditions in all maternity hospitals, with a friendlier environment, with freedom of movement, with feeding, with the presence of a loved one of the pregnant woman who has received prenatal training, and finally, with the timely information of the pregnant woman by making a decision of an EMCS, as well as providing psychological support perioperatively. Special care should be taken in the postpartum period of women whose birth experience has been recorded as a traumatic experience by the mother. Therefore, midwifery psychoeducation training from the first weeks of pregnancy must aim at the mental empowerment of women and their partners in general so that they are able to cope with the transition to parenthood, especially if it comes through mental trauma. The present proposals to health professionals aim to supplement and improve the already existing methods.

## Figures and Tables

**Table 1:** Chi-squared test of type of CS in relation to diagnosis.

	EMCS		ELCS		
Diagnosis	*N*	%	*N*	%	*p*
No Diagnosis	114	62.98	264	91.67	*p* < 0.001
PTSD Profile	20	11.05	16	5.56	
PTSD	47	25.97	8	2.79	

**Table 2:** Perinatal and mental health variables in relationship to PTSD.

Perinatal and Mental Health Variables	f/M	rf/SD	*p*
Gestational Week	37.76	2.10	*p* < 0.001 ^b^
<37	70	14.9	
≥37	399	85.1	
Total	469	100.0	
Birth Expectations/Satisfaction			*p* < 0.001 ^b^
No	245	52.2	
Yes	224	47.8	
Total	469	100.0	
Psychiatric History			*p* < 0.001 ^b^
No	411	87.6	
Yes	58	12.4	
Total	469	100.0	
Traumatic CS			*p* < 0.001 ^b^
No	230	49.0	
Yes	239	51.0	
Total	469	100.0	
Criterion A1—Was your life or your child’s life in danger?			*p* < 0.001 ^b^
No	132	56.7	
Yes, My Child	63	27.0	
Yes, Mine	17	7.3	
Yes, Both of Us	21	9.0	
Total	233	100.0	
Criterion A2—Any complications involving you or your child?			*p* < 0.001 ^b^
No	161	70.0	
Yes, My Child	43	18.7	
Yes, Me	16	7.0	
Yes, Both of Us	10	4.3	
Total	230	100.0	

Note. ^b^—ANOVA.

**Table 3:** Linear regression of four LEC-5 categories of response in relation to PTSD.

LEC-5 Categories of Response	b	S.E.	β	t	*p*
(Constant)	4.79	0.75		6.35	*p* < 0.001
Direct Exposure	4.39	0.45	0.41	9.68	*p* < 0.001
Witness to the Event	4.48	1.12	0.17	4.00	*p* < 0.001
Information of the Event	−0.30	0.79	−0.02	−0.38	ns
Exposure in the Working Space	−1.40	2.23	−0.03	−0.63	ns

Note. R = 0.228, R^2^ = 0.222, F = 34.35, df = 4, *p* < 0.001, ns = not statistically significant.

**Table 4:** Linear regression of PTSD in relation to 17 LEC-5 traumatic events.

	b	S.E.	β	t	*p*
(Constant)	4.74	0.76		6.28	*p* < 0.001
1. Natural Disaster	−1.57	2.62	−0.03	−0.60	ns
2. Fire/Explosion	3.25	2.70	0.05	1.20	ns
3. Transportation Accident	0.94	2.01	0.02	0.47	ns
4. Serious Accident at Work/Home/During Recreation	4.83	2.89	0.07	1.67	ns
5. Exposure to Toxic Substance	−0.56	5.40	−0.01	−0.10	ns
6. Physical Assault	6.62	2.40	0.13	2.76	0.006
7. Assault with a Weapon	19.17	4.66	0.20	4.11	0.039
8. Sexual Assault	5.47	3.57	0.07	1.53	ns
9. Unwanted Sexual Experience	2.99	3.24	0.04	0.92	ns
10. Combat/Exposure to a Warzone	−2.35	12.08	−0.02	−0.19	ns
11. Captivity	2.26	9.08	0.02	0.25	ns
12. Life-Threatening Illness/Injury	5.35	2.42	0.09	2.22	0.027
13. Severe Human Suffering	6.49	1.58	0.18	4.11	*p* < 0.001
14. Sudden Violent Death	12.99	3.50	0.16	3.72	0.007
15. Sudden Accidental Death	−0.79	2.19	−0.02	−0.36	ns
16. Serious Injury/Harm/Death You Caused to Someone	−1.02	7.26	−0.01	−0.14	ns
17. Other Very Stressful Experience	4.90	1.93	0.11	2.54	0.011

Note. R = 0.292, R^2^ = 0.265, F = 10.94, df = 17, *p* < 0.001, ns = not statistically significant.

**Table 5:** Linear regression of PTSD profile in relation to 17 LEC-5traumatic events.

	b	S.E.	β	t	*p*
(Constant)	0.03	0.02		1.94	ns
1. Natural Disaster	0.01	0.06	0.01	0.16	ns
2. Fire/Explosion	0.15	0.06	0.12	2.55	0.011
3. Transportation Accident	0.00	0.05	0.00	0.01	ns
4. Serious Accident at Work/Home/During Recreation	0.17	0.08	0.11	2.23	0.026
5. Exposure to Toxic Substance	0.13	0.13	0.05	0.98	ns
6. Physical Assault	−0.05	0.06	−0.04	−0.77	ns
7. Assault with a Weapon	0.59	0.14	0.23	4.35	*p* < 0.001
8. Sexual Assault	0.19	0.09	0.11	2.14	0.033
9. Unwanted Sexual Experience	0.11	0.07	0.07	1.49	ns
10. Combat/Exposure to a Warzone	−0.17	0.27	−0.04	−0.61	ns
11. Captivity	0.47	0.18	0.16	2.59	0.010
12. Life-Threatening Illness/Injury	0.21	0.06	0.17	3.62	*p* < 0.001
13. Severe Human Suffering	0.05	0.04	0.07	1.39	ns
14. Sudden Violent Death	0.10	0.09	0.06	1.15	ns
15. Sudden Accidental Death	0.04	0.05	0.04	0.84	ns
16. Serious Injury/Harm/Death You Caused to Someone Else	−0.03	0.21	−0.01	−0.15	ns
17. Other Very Stressful Experience	0.07	0.04	0.08	1.60	ns

Note. R = 0.217, R^2^ = 0.184, F = 6.46, df = 17, *p* < 0.001, ns = not statistically significant.

## Data Availability

Not applicable.
